# INSAF-HAS: a tool to select patients with hypertension for pharmaceutical care

**DOI:** 10.31744/einstein_journal/2020AO4858

**Published:** 2019-12-06

**Authors:** Beatriz Maria Pereira Girolineto, Alan Maicon de Oliveira, Ana Maria Rosa Freato Gonçalves, Marília Silveira de Almeida Campos, Leonardo Régis Leira Pereira

**Affiliations:** 1 Universidade Federal do Piauí, Teresina, PI, Brazil.; 2 Faculdade de Ciências Farmacêuticas de Ribeirão Preto, Universidade de São Paulo, Ribeirão Preto, SP, Brazil.

**Keywords:** Pharmaceutical services, Hypertension, Antihypertensive agents, Surveys and questionnaires, Triage, Professional-patient relations, Quality management, Patient selection

## Abstract

**Objective:**

To develop and validate the content of a tool aimed to select patients with hypertension for pharmaceutical care, based on identification of individuals in greater need of attention.

**Methods:**

The tool was developed and assessed for face and content validity, which was carried out in three stages. Phase I consisted of comprehensive literature review, which prompted the development of the first version of the tool. Phase II consisted of validation by an expert panel. Phase III consisted of a pilot study with hypertensive patients and preparation of the final version of the instrument.

**Results:**

Literature review yielded 30 studies, out of which 13 factors associated with hypertension and cardiovascular disease control and complications were selected. Once the initial version of the tool named INSAF-HAS was obtained, four expert meetings were held, each leading to instrument improvement until a final consensus was reached. In the pilot study, INSAF-HAS was applied to 30 patients with a diagnosis of hypertension for applicability pretest; adjustments were made and the final version of INSAF-HAS obtained.

**Conclusion:**

The INSAF-HAS tool developed in this study has face and content validity, and may contribute to the selection of patients with hypertension in greater need of pharmaceutical care services.

## INTRODUCTION

Hypertension (HTN) is a multifactorial clinical condition characterized by sustained elevation of blood pressure levels, with potential injury to target organs.^1^ The 2010 prevalence of hypertension in medium- and low-income countries, such as Brazil, and in high-income countries amounted to 31.5% (30.2% to 32.9%) and 28.5% (27.3% to 29.7%), respectively.^2^ According to research data, the prevalence of HTN in the Brazilian population ranges from 22.3% to 64.1%.^3 - 9^

Uncontrolled HTN may trigger the development and/or aggravation of cardiovascular diseases, such as stroke, acute myocardial infarction (MI) and heart failure.^10^ Therefore, HTN treatment is vital to prevent these events.^10^

Pharmacotherapy plays an important role in HTN management; however, evidence suggests that blood pressure control among hypertensive patients is poor, with only 15.5% to 63% achieving that goal.^11 - 15^ Several factors may contribute to pharmacotherapy non-effectiveness, such as medication errors, inadequate prescription and lack of adherence treatment, among others.

Effective contribution of clinical pharmacists to improvement of patient quality of life, identification and resolution of pharmacotherapeutic problems, control of clinical parameters and reduction of costs associated with chronic diseases, such as *diabetes mellitus* (DM), dyslipidemia and HTN has been consistently demonstrated in several studies.^16 - 21^ Hence, integration of pharmaceutical care^22^ with health care provision is an effective strategy, with potential clinical, humanistic and economic impacts.^23 - 30^

In Brazil, pharmacists are often deeply involved in management activities, making it difficult to provide clinical advice for patients requiring personalized attention.^31 - 33^ Triage tools critically related to successful clinical pharmacy practice are needed to expand and direct this approach to patients with greater pharmacotherapeutic needs.^34^

## OBJECTIVE

To develop and validate a tool aimed to select patients suffering from hypertension with greater therapeutic needs for inclusion in pharmaceutical care services.

## METHODS

**Figure 1:**
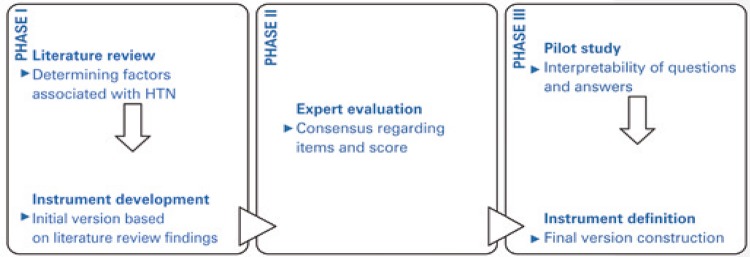
Tool development process based on face and content validity HTN: hypertension.

This study involved three phases of development and validation of a screening tool designed for HTN patients (INSAF-HAS; Figure 1 ). Phase I consisted of a literature review for preparation of the initial version of the tool. In phase II, the instrument was evaluated by an expert panel. Phase III consisted of a pilot to test respondents’ understanding of questions and questionnaire practicality in order to define the final version. This study was approved by the Research Ethics Committee of *Faculdade de Ciências Farmacêuticas de Ribeirão Preto, Universidade de São Paulo* , protocol no. 67/2011.

### Phase I: literature review and instrument development

A literature review was carried out to identify factors associated with HTN. PubMed^®^ database was searched using Medical Subject Headings (MeSH) terms related to pharmaceutical care (pharmaceutical services), HTN (hypertension and risk factors) and adherence to medical therapy (medication adherence and patient compliance).

Following article selection, references related to the topic listed in the first studies were also examined. The initial version of INSAF-HAS was then created, based on critical reading of selected documents.

### Phase II: expert panel

The first version was evaluated by five expert pharmacists with a solid background (teaching, research and professional experience) in pharmaceutical care provision and HTN, all of them PhD students in this field.^35^ Expert panels consisted of in-person meetings for analysis of individual item pertinence and clarity. Experts could also refine concepts and suggest inclusion of elements potentially associated or not associated with HTN control.

### Phase III: pilot study and definition of the official instrument

A pilot study was then carried out for identification of potential difficulties associated with respondents’ understanding of questions and questionnaire practicality and applicability. This phase was conducted at a primary care unit located in the city of Ribeirão Preto (SP), Brazil. In order to minimize potential questionnaire biases, a duly trained pharmacist applied the instrument (face-to-face interviews) to a convenience sample of patients diagnosed with HTN aged 18 years or older, and using at least one anti-hypertensive drug. Volunteers were recruited at the pharmacy of the aforementioned primary care unit while getting their prescriptions.

Difficulties noted were discussed and items rephrased or modified according to expert consensus view. Pilot study completion was achieved when 11 consecutive participants reported and revealed proper understanding of instrument items.

The final version was then prepared and item weights assigned according to experts’ opinion to yield a final score.

## RESULTS

### Phase I

Thirty studies (including guidelines) were selected; these were analyzed for definition of indicators to be included in the instrument. Following critical review, factors reported in literature as being associated with blood pressure (BP) control, HTN- and cardiovascular disease-related complications and aggravations, adherence to pharmacological treatment and occurrence of pharmacotherapy-related problems were selected ( Table 1 ). Nonetheless, some controversial factors, such as stress, depression and coffee intake were not included.

**Table 1:** Determining factors in clinical outcomes related to progression of hypertension

Factors	Studies
Age and sex	*Sociedade Brasileira de Cardiologia* ^(36)^
	Gueyffier et al.^(37)^
	Takahashi et al.^(38)^
Level of education	Vasconcelos et al.^(39)^
Regular physical exercise practice	*Sociedade Brasileira de Cardiologia* ^(36)^
	O´Donnell et al.^(40)^
	Pescatello et al.^(41)^
Smoking	*Sociedade Brasileira de Cardiologia* ^(36)^
	Gueyffier et al.^(37)^
	O´Donnell et al.^(40)^
	Ridker et al.^(42)^
	Rempher^(43)^
Alcohol abuse	*Sociedade Brasileira de Cardiologia* ^(36)^
	Parekh et al.^(44)^
	Taylor et al.^(45)^
Adherence to pharmacotherapy	Vasconcelos et al.^(39)^
	Bunker et al.^(46)^
	Balkrishnan^(47)^
Access to drugs	Vasconcelos et al.^(39)^ Balkrishnan^(47)^
Need of care provider	Vasconcelos et al.^(39)^
Use of drugs potentially leading to BP elevation	*Sociedade Brasileira de Cardiologia* ^(36)^
	Amer et al.^(48)^
	Snowden et al.^(49)^
Comorbidities: *diabetes mellitus* and dyslipidemia	Gueyffier et al.^(37)^
	Ridker et al.^(42)^
	Piegas et al.^(50)^
	Cubeddu et al.^(51)^
	Reaven.^(52)^
Previous aggravations: acute MI and stroke	Gueyffier et al.^(37)^
	Schmieder^(53)^
Family history: HTN, dyslipidemia, *diabetes mellitus,* acute MI and stroke	Harrison et al.^(54)^
	Kennedy et al.^(55)^

BP: blood pressure; MI: myocardial infarction; HTN: hypertension.

The initial version of INSAF-HAS ( Appendix 1 ) was then obtained. Over the course of assessments, factors potentially affecting practical application of the instrument and response reliability (obesity, salt intake, previous renal failure or hypertensive retinopathy) were also excluded.

### Phase II

**Figure 2:**
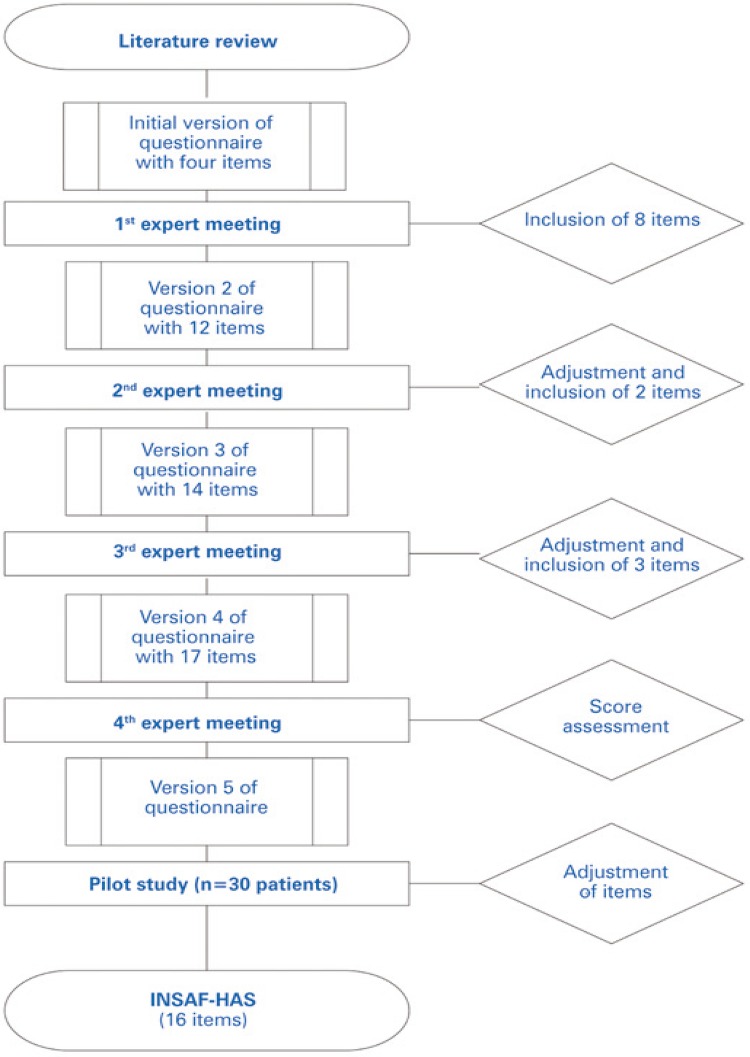
Flowchart displaying INSAF-HAS development process

Four expert meetings were required for analysis and discussion until a final consensus was reached ( Figure 2 ). Item clarity and relevance of each indicator were assessed during such meetings; individual item weights and final scores were then attributed accordingly. Each meeting led to modifications of the tool (versions 2 to 5) ( Appendix 1 ).

Different weights were attributed to different factors according to their respective significance for clinical progression of HTN. A score ranging from 9 to 200 ( Table 2 ) was then defined; the lower the score, the lower the need of pharmaceutical care.

**Table 2:** Minimum and maximum scores per INSAF-HAS item

Questionnaire item	Minimum score	Maximum score
1	1	9
2	0	8
3	0	6
4	0	5
5	0	13
6	0	9
7	0	7
8	0	11
9	0	9
10	8	23
11	0	7
12	0	16
13	0	7
14	0	16
15	0	52
16	0	2
**Total**	9	200

### Phase III

After the expert panel reached a final consensus about INSAF-HAS, a pre-test procedure was put in place to test the applicability of the instrument (i.e., to check whether respondents were actually able to understand questions). Thirty patients diagnosed with HTN were approached while getting their prescriptions at the primary care unit, until a total of 11 consecutive participants revealed proper understanding of all instrument items.

The questionnaire was further modified during this period ( Table 3 ). Some questions were rephrased for improved interpretability; these formed the final INSAF-HAS version ( Appendix 2 ).

**Table 3:** Modifications introduced in INSAF-HAS after completion of the pilot study

Version 5 of INSAF-HAS	Reason for change	Final version of INSAF-HAS
What is your level of education (how long did you study)?	Difficulties in speech interpretation	What is your level of education (up to what grade did you study)?
Do you consume alcoholic drinks?	More objective question to assist interviewer	Do you consume alcoholic drinks? If yes, how much?
Items 6 to 9 (comorbidities)	Order changed to facilitate prescription analysis	New order: items 11 to 14
Dot you have high blood pressure?	Many patients were not able to describe their blood pressure control status; this led to doubts, as all had been diagnosed with HTN	Exclusion
If answer to question 10 was “yes”, how do you treat your high blood pressure problem?	Changes due to exclusion of item 10	How do you treat your high blood pressure problem?
Items 12 and 13 (HTN aggravations and family history, respectively)	Order changed to facilitate prescription analysis and improve patient interview dynamics	New order: items 15 and 16
Items 14, 15 and 16 (adherence and access to medication)	Order changed to facilitate prescription analysis and improve patient interview dynamics	New order: items 6, 7 and 9, respectively

## DISCUSSION

This study led to the development of a dedicated tool for analysis of factors associated with therapeutic and clinical aspects of HTN patients. Scientific evidences supporting screening tools for pharmaceutical care users are scarce and most studies approach different pharmacotherapeutic and clinical issues without a thorough, holistic assessment of patient profile.^56 - 60^ The fact that INSAF-HAS is not an inclusion or exclusion tool, but rather an instrument aimed to indicate individual priority of care, must be emphasized. Therefore, once successful care of selected patients is achieved, other patients meeting priority criteria may receive personalized pharmaceutical care.

Current health system demands emphasize the significance of clinical and public health education services provided by pharmacists.^61 , 62^ Still, motivation levers encouraging practical application of pharmaceutical knowledge are lacking, since the technicist profile prevails in several services (particularly in low- and middle-income countries). Moreover, there is outpatient health care overcrowding and ineffective management. These factors translate into lack of follow-up in patient-centered care.^62 , 63^ Aside from management and technical activities, tools aimed to optimize pharmaceutical care and promote inclusion of equitable access to pharmaceutical services in emerging initiatives, such as management of chronic non-communicable diseases and reduction in their mortality rates, are needed.^62^

This is a complex scenario involving organizational aspects of health care services and compatible professional expertise; up-front provision of pharmaceutical care to high risk patients may contribute to improved management of professional procedures and benefit patients in urgent need of critical care.^64^

The reality faced by pharmacists in charge of drug dispensing was taken into account in INSAF-HAS development, for these professionals will be able to use this tool in daily routine. Given the wide range of activities undertaken (care provision to patients, drug acquisition, inventory control and management-related activities), a simple, user-friendly questionnaire amenable to application within a limited time frame was developed. Complex, time-consuming procedures would hardly fit into the busy routine of pharmacists. INSAF-HAS comprises factors driving clinical progression of HTN that can be easily interrogated in the presence of affected patients ( *e.g* ., during drug dispensing) and constitutes an important aid in selection of patients for pharmaceutical care; it is also amenable to application by pharmacy technicians and assistants.

Among factors considered for instrument construction, elevated BP stands out as a significant risk factor for cardiovascular disease development and/or aggravation.^36 , 37 , 42 , 65 , 66^ However, BP measurement may limit INSAF-HAS application by pharmacists and pharmacy teams due to the time required for completion of this procedure. Instead, experts discussed the possibility of including one question interrogation blood pressure control. Given blood pressure may not be measured frequently enough ( *i.e* ., patients may be not be aware of their BP levels), such a question might introduce a bias in patients selection. Therefore, questions interrogating blood pressure monitoring were not included. However, the tool is aimed at patients suffering from HTN, a risk factor for cardiovascular diseases *per se* .^40 , 67^ In an effort to improve instrument content and applicability, experts agreed upon the inclusion of one item interrogating BP treatment.

Elevated serum cholesterol (total and subfractions) is another important risk factor for cardiovascular disease.^42 , 50^ In Brazilian public laboratory services, this parameter is measured approximately every 6 months. Ideally, cholesterol levels should be measured via lab tests; however, given this may not be feasible due to time and financial constraints, values (potentially not up-to-date) would have to be extracted from medical records or new tests requested. Instead, questions interrogating dyslipidemia and respective treatment, backed by prescriptions, were thought to be a better alternative.

*Diabetes mellitus* , also a risk factor for cardiovascular disease,^37 , 42 , 51 , 52^ was assessed in a similar manner to dyslipidemia. Instead of resorting to laboratory (blood glucose or glycated hemoglobin) or capillary blood glucose test (finger pricking), DM was interrogated and confirmed via medical prescription of oral anti-diabetic drugs or insulin, ensuring INSAF-HAS accessibility and practicality.

Smoking, yet another risk factor for cardiovascular disease,^36 , 37 , 40 , 42 , 43^ was interrogated in INSAF-HAS via the direct question: “Have you ever smoked/do you currently smoke cigarettes?”. The word “smoker” alone was avoided because it may be associated with illicit drug use (for instance, marijuana or crack), according to experts. “Cigarette smoking” was thought to be a more familiar term, particularly among individuals with lower levels of education, and was therefore selected for inclusion in INSAF-HAS.

Although some studies suggest beneficial effects of small doses of alcohol, red wine in particular, for atherosclerosis,^68 , 69^ alcohol consumption is associated with increased risk of HTN and should be avoided.^44 , 45^ According to the Brazilian Hypertension Guidelines [ *Diretrizes Brasileiras de Hipertensão* ], daily alcohol consumption should not exceed 30g or 15g (men and women, respectively).^36^ Therefore, these levels of alcohol consumption were interrogated in INSAF-HAS.

Other important factors included in the instrument were patient´s age and sex.^36 - 38^ Male individuals are more prone to developing cardiovascular disease, particularly after the age of 55 years. In women, this risk increases after the onset of menopause (approximately at 50 years) and tends to rise even more after the age of 65 years.

Physical exercise practice reduces the risk of cardiovascular diseases, whereas sedentarism is a predisposing factor.^36 , 40 , 41^ Therefore, the tool proposed in this study interrogates physical exercise practice (physical activities lasting at least 30 minutes, three times per week, or 90 minutes/week).

Family history of HTN, dyslipidemia, DM and some conditions, such as acute MI and stroke in first-degree relatives, are also thought to be risk factors for cardiovascular diseases.^54 , 55^ One question interrogating family history was therefore included in INSAF-HAS.

Previous HTN-related conditions, such as stroke, acute MI, renal failure and hypertensive retinopathy must also be accounted for.^37 , 53 , 70^ Given their significance, these factors were included in INSAF-HAS; however, during the pre-test phase, respondents were often unable to provide reliable information regarding such aggravations.

Some patients were not able to understand the meaning of the term “renal failure”. The term “kidney problem” was then selected, but lack of understanding persisted. When asked whether they “had kidney problems”, patients were not able to differentiate between renal failure and other diseases affecting the urinary system, such as cystitis or nephrolithiasis. As use of medical record data was not an option, and reliable confirmation of renal failure diagnosis could not be obtained via self-reporting, this cardiovascular disease risk factor was excluded from the final version of the instrument.

Likewise, most patients were unable to grasp the meaning of “hypertensive retinopathy” in the pre-test phase. The term “vision problems” was then selected, but disorders associated with myopia and cataract induced patients to answer “yes”, when interrogated about their “type of vision problem”, precluding reliable data collection. Since the diagnosis could not be confirmed, this item was excluded from the final version of INSAF-HAS to mitigate potential errors.

Different from aforementioned aggravations, stroke and acute MI were easily self-reported. Patients had no doubts regarding the questions: “Have you ever had an infarction?” and “Have you ever had a cerebrovascular accident (CVA) or stroke?”. Therefore, these items were retained in the instrument.

As regards pharmacotherapy, poor adherence to prescribed anti-hypertensive treatment is associated with poor BP control.^46^ Several aspects potentially interfering with compliance to plan of care, such as level of education (the lower the level of education, the greater the difficulty in properly understanding and following prescriptions), access to medications, ability to self-medicate and polypharmacy were accounted for.^39 , 47^ Individuals were also directly interrogated about adherence ( *i.e* ., whether they have ever forgotten/chosen not to take medications as prescribed).

Controversial HTN-related factors, such as stress,^67 , 71^ depression^67 , 72^ and coffee intake^36 , 73^ were not included. Obesity and salt intake^36 , 40 , 74 - 76^ were considered, but were also estimated to negatively interfere with tool application. In the case of obesity, time constraints associated with weight and height measurements required for body mass index calculation were the major reasons for exclusion. As regards salt intake, daily amounts consumed were deemed difficult to measure, as salt is not the only source of sodium in foods and people are often unable to quantify their daily salt consumption.

### Study limitations and strengths

Assessment of some risk factors for cardiovascular disease was not consistent with tool objectives (accessibility and feasible application in routine pharmacy settings); these were therefore not included in the final version. However, INSAF-HAS is aimed to triage patients who will then receive pharmaceutical care; hence, excluded factors may still be assessed over the course of pharmaceutical care provision.

The fact that expert panel members selected to evaluate INSAF-HAS were all staff at the *Faculdade de Ciências Farmacêuticas de Ribeirão Preto* , *Universidade de São Paulo* , is a limitation of this study.

Detailed description of item selection criteria, proper definition of the target population and conduction of a pilot study supported INSAF-HAS face and content validity. However, future studies assessing psychometric properties are needed.

In spite of these limitations, this initial study may provide significant contributions to the structuring of pharmaceutical care services. INSAF-HAS may be used to screen for patients in greater need of care, thereby optimizing professional care provision. This study may also encourage related debate and support pharmaceutical care practices in Brazil.

## CONCLUSION

The INSAF-HAS tool created has face and content validity; it also lists and scores risk factors for hypertension among affected individuals. This tool may be used to screen patients in greater need of pharmaceutical care.
