# Supplementary material for: INSAF-HAS: a tool to select patients with hypertension for pharmaceutical care
Source: Einstein (Sao Paulo). 2019 Dec 6;18:eAO4858. doi: 10.31744/einstein_journal/2020AO4858 (PMC6905168; doi:10.31744/einstein_journal/2020AO4858)
Supplement: Supplementary file 1 [file 2317-6385-eins-18-eAO4858-Suppl01.pdf]

APPENDIX 1

Initial version of INSAF-HAS

Name:____

Record No.:________ Sex:________ Date of birth: ___/___/____ Age:________

1) Risk factors and associated diseases

Blood pressure (mean) [/] mmHg

BMI (weight/height^2):

Value [ ]

☐ Obese (severity______)

☐ Overweight

☐ Normal

Smoker (indicate with x) *:

☐ Yes

☐ No

☐ Former smoker

Dyslipidemia (high cholesterol)*:

☐ Yes

☐ No

Alcoholic drink consumption *:

☐ Yes (frequency__________)

☐ No

Diabetic*:

☐ Yes

☐ No

Physical activity*:

☐ Yes (frequency__________)

☐ No

2) Family history (tick up to second-degree relative)*

☐ Hypertension

☐ Dyslipidemia

☐ Diabetes

☐ Visual impairment**

☐ Cardiovascular disease

☐ Stroke

☐ Renal failure

3) Complications (tick)*

☐ Cardiovascular disease

☐ Stroke

☐ Renal failure

☐ Visual impairment**

4) Number of drugs taken:

Total		Anti-hypertensive	

* Self-reported

** Hypertension-related

Second version of INSAF-HAS

Name:____

Address:____

Telephone: (__)__________________ Mobile: (__)__________________

Record No.:_________________ Date of birth:____ /______/______

1) Age and gender:

☐ Female, 65 years or less

☐ Female, over 65 years

☐ Male, 55 years or less

☐ Male, over 55 years

Tick patient's answer to the following questions:

continue...

...Continuation

APPENDIX 1

2) Do you practice physical activities? (If yes, consider only frequency as indicated in answer)1:

☐ Yes, three times per week or more, for at least 30 minutes (90 minutes/week)

☐ No

3) Do you consume alcoholic drinks?[2]

Woman:	Man:
☐ Yes, more than 15g in a single day	☐ Yes, more than 30g in a single day
☐ No	☐ No

4) Do you smoke cigarettes?

☐ Yes

☐ Former smoker

☐ No

5) Do you have high cholesterol?

☐ Yes

☐ No

6) Are you diabetic?

☐ Yes

☐ No

7) Do you have high blood pressure?

☐ Yes

☐ No

[1] Consider "physical activity" sports practices, such as walking, running, swimming and others. Disregard household activities, such as brooming, washing, etc.

[2] Consider alcohol consumption in a single day. If patient consumes less than 30g (men) or 15g (women) every day, mark "no". If consumption exceeds amounts indicated one or more days per week, tick "yes". Thirty grams of alcohol correspond to approximately two cans or bottles of beer, two glasses (150mL) of wine or two doses (50mL) of spirits (whisky, vodka and others).

8) Do you have/have you ever had?

☐ Infarction

☐ Stroke or CVA

☐ Kidney problems

☐ Vision problems[3]

9) In your family, has any of your relatives (up to second degree) ever had:

☐ High blood pressure

☐ High cholesterol

☐ Diabetes

☐ Went blind

☐ Infarction

☐ Stroke or CVA

☐ Renal problems

10) Have you ever forgotten/chosen not to take any of the drugs prescribed to you by your physician:

☐ Yes

☐ No

11) Can you obtain all drugs prescribed to you by your physician here at the primary care unit pharmacy? If not, how do you get the remaining ones?[4]

☐ Yes

☐ No, I have, buy or am given some

☐ No, I cannot obtain some

12) Number of drugs taken[5]:

☐ Drugs for blood pressure

☐ Drugs for diabetes

☐ Drugs for cholesterol

☐ Drugs associated with HTN aggravation[6]

☐ Others

☐ Total

continue...

...Continuation

APPENDIX 1

[3] Do not consider myopia, hyperopia, astigmatism and others not secondary to systemic arterial hypertension.

[4] Check whether patient uses drugs other than those prescribed.

[5] Consider drugs prescribed other than those listed in prescriptions presented.

[6] Such as: immunosuppressants (cyclosporine and tacrolimus), anorexigenic drugs/satiety agents (amfepramone and others), anti-neoplastic agents (bevacizumab, estramustine, gemtuzumab ozogamicin and pazopanib), antidepressants (tricyclic and monoamine oxidase inhibitors), chronic use of corticosteroids, non-steroidal anti-inflammatory drugs, cyclooxygenase 1 and cyclooxygenase 2 inhibitors.

Third version of INSAF-HAS

Name:____

Address:____

Telephone: (___)___________ Mobile: (___)_________ Date: _____/_____/_______

Record No.:______________ Date of birth:________/________/________

1) What is your age and gender (sex)?

☐ Female, 49 years or less

☐ Female, 50 to 65 years

☐ Female, over 65 years

☐ Male, 55 years or less

☐ Male, over 55 years

2) What is your level of education (how long did you study)?

☐ Illiterate

☐ Primary education, incomplete

☐ Primary education, complete

☐ Midschool incomplete

☐ Midschool complete

☐ Higher education, incomplete

☐ Higher education, complete

3) Do you practice physical exercise? (If yes, consider only frequency above or equal to that indicated in answer)[1]:

☐ Yes, three times per week or more, for at least 30 minutes (90 minutes/week)

☐ No

4) Do you consume alcoholic drinks?[2]

a) If female patient:

☐ Yes, more than 15g in a single day

☐ No

b) If male patient:

☐ Yes, more than 30g in a single day

☐ No

5) Do you smoke cigarettes?

☐ Yes

☐ Former smoker

☐ No

6) Do you have high cholesterol?

☐ Yes

☐ No

7) Are you diabetic?

☐ Yes

☐ No

8) Do you have high blood pressure?

☐ Yes

☐ No

[1] Consider "physical activity" sports practices, such as walking, running, swimming and others. Disregard household activities such as brooming, washing, etc.

[2] Consider alcohol consumption in a single day and check patient gender in answer. If patient consumes less than 30 g (men) or 15 g (women) every day, tick "no". ". If consumption exceeds amounts indicated one or more days per week, tick "yes". Thirty grams of alcohol correspond to approximately two cans or bottles of beer, two glasses (150 mL) of wine or two doses (50 mL) of spirits (whisky, vodka and others).

continue...

...Continuation

APPENDIX 1

9) Do you have/have you ever had?
- [ ] Infarction
- [ ] Stroke or CVA
- [ ] Kidney problems
- [ ] Vision problems[3]

10) In your family, has any of your relatives (up to second degree) ever had:
- [ ] High blood pressure
- [ ] High cholesterol
- [ ] Diabetes
- [ ] Went blind
- [ ] Infarction
- [ ] Stroke or CVA
- [ ] Kidney problems

11) Have you ever forgotten/chosen not to take any of the drugs prescribed to you by your physician?
- [ ] Yes
- [ ] No

12) Do you need help to take your medications?
- [ ] Yes
- [ ] No

13) Can you obtain all drugs prescribed to you by your physician here at the primary care unit pharmacy? If not, how do you get the remaining ones?[4]
- [ ] Yes
- [ ] No, I have, buy or am given some.
- [ ] No, I cannot obtain some

14) Patient currently uses[5]:
- [ ] Drugs for blood pressure
- [ ] Drugs for diabetes
- [ ] Drugs for cholesterol
- [ ] Drugs associated with HTN aggravation[6]
- [ ] Others

[3] Do not consider myopia, hyperopia or astigmatism.

[4] Check whether patient uses drugs other than those prescribed.

[5] Consider drugs prescribed other than those listed in prescriptions presented. Mark drugs classes used by patients and listed in answer.

[6] Such as: immunosuppressants (cyclosporine and tacrolimus), anorexigenic drugs/satiety agents (amfepramone and others), anti-neoplastic agents (bevacizumab, estramustine, gemtuzumab ozogamicin and pazopanib), antidepressants (tricyclic and monoamine oxidase inhibitors), chronic use of corticosteroids, non-steroidal anti-inflammatory drugs, cyclooxygenase 1 and cyclooxygenase 2 inhibitors.

Fourth version of INSAF-HAS

Name:____

Address:____

Telephone: (___)_____________ Mobile: (___)_________ Date: _____/_____/_______

Record No.:______________ Date of birth:________/________/________

1) What is your age and gender (sex)?
- [ ] Female, 49 years or less
- [ ] Female, 50 to 65 years
- [ ] Female, over 65 years
- [ ] Male, years or less
- [ ] Male, over 55 years

2) What is your level of education (how long did you study)?
- [ ] Illiterate
- [ ] Primary education, complete
- [ ] Middle school, complete
- [ ] Higher education, complete

continue...

...Continuation

APPENDIX 1

3) Do you practice physical exercise? (If yes, consider only frequency above or equal to that indicated in answer)[1]:

☐ Yes, three times per week or more, for at least 30 minutes (90 minutes/week)

☐ No

4) Do you consume alcoholic drinks?[2]

a) If female patient:

☐ Yes, more than 15g in a single day

☐ No

b) If male patient:

☐ Yes, more than 30g in a single day

☐ No

5) Do you smoke cigarettes?

☐ Yes

☐ Former smoker

☐ No

6) Do you have high cholesterol?

☐ Yes

☐ No

7) If you answered yes to question 6, how do you treat your high cholesterol problem?

☐ With diet

☐ With medication

☐ No treatment was prescribed/do not treat

8) Are you diabetic?

☐ Yes

☐ No

[1] Consider "physical activity" sports practices such as walking, running, swimming and others. Disregard household activities such as brooming, washing, etc.

[2] Consider alcohol consumption in a single day and check patient gender in answer. If patient consumes less than 30g (men) or 15g (women) every day, tick "no". If consumption exceeds amounts indicated one or more days per week, tick "yes". Thirty grams of alcohol correspond to approximately two cans or bottles of beer, two glasses (150mL) of wine or two doses (50mL) of spirits (whisky, vodka and others).

9) If you answered yes to question 8, how do you treat your diabetes problem?

☐ With diet

☐ With medication

☐ No treatment was prescribed/do not treat

10) Do you have high blood pressure?

☐ Yes

☐ No

11) If you answered yes to question 10, how do you treat your high blood pressure problem?

☐ With diet

☐ With medication

☐ No treatment was prescribed/do not treat

12) Have you ever had/ do you currently have:

☐ Infarction

☐ Stroke or CVA

☐ Kidney problems

☐ Vision problems[3]

13) In your family, has any of your relatives (up to first degree[4]) ever had:

☐ High blood pressure

☐ High cholesterol

☐ Diabetes

☐ Went blind

☐ Infarction

☐ Stroke or CVA

☐ Kidney problems

continue...

...Continuation

APPENDIX 1

14) Have you ever forgotten/chosen not to take any of the drugs prescribed to you by your physician?

☐ Yes

☐ No

15) Do you need help to take your medications?

☐ Yes

☐ No

16) Can you obtain all drugs prescribed to you by your physician here at the primary care unit pharmacy? If not, how do you get the remaining ones?[5]

☐ Yes

☐ No, I have, buy or am given some

☐ No, I cannot obtain some

17) Patient currently uses[6]:

☐ Drugs associated with HTN aggravation[7]

☐ Others[8]

[3] Do not consider myopia, hyperopia or astigmatism.

[4] Consider only parents and siblings.

[5] Check whether patient uses drugs other than those prescribed.

[6] Consider drugs prescribed other than those listed in prescriptions presented.

[7] Such as: immunosuppressants (cyclosporine and tacrolimus), anorexigenic drugs/satiety agents (amfepramone and others), anti-neoplastic agents (bevacizumab, estramustine, gemtuzumab ozogamicin and pazopanib), antidepressants (tricyclic and monoamine oxidase inhibitors), chronic use of corticosteroids, non-steroidal anti-inflammatory drugs, cyclooxygenase 1 and cyclooxygenase 2 inhibitors.

[8] Disregard antidyslipidemic, antidiabetic and antihypertensive drugs included in questions 7, 9 and 11, respectively.

Fifth version of INSAF-HAS

Name:____

Address:____

Telephone: (___)____________Mobile: (___)_________ Date: _____/_____/_______

Record No.:______________ Date of birth:______ /_______/________

1) What is your age and gender (sex)?

☐ Female, 49 years of less

☐ Female, 50 to 64 years

☐ Female, 65 years of older

☐ Male, 54 years of less

☐ Male, 55 years of older

2) What is your level of education (how long have you studied)[1]?

☐ Illiterate

☐ Primary education (1st stage of education)

☐ Middle school (2nd stage or high school) or higher education

3) Do you practice physical exercise? (If yes, consider only frequency above or equal to that indicated in answer)[2]:

☐ Yes, three times per week or more, for at least 30 minutes (90 minutes/week)

☐ Não

4) Do you consume alcoholic drinks?[3]

a) If female patient:

☐ Yes, more than 15g in a single day

☐ No

b) If male patient:

☐ Yes, more than 15g in a single day

☐ No

5) Do you smoke cigarettes?

☐ Yes

☐ Former smoker

☐ No

6) Do you have high cholesterol?

☐ Yes

☐ No

continue...

...Continuation

APPENDIX 1

7) If yes to question 6, how do you treat your high cholesterol problem?

☐ With diet

☐ With drugs

☐ No treatment was prescribed/do not treat

[1] Regardless of completion of a given stage, *e.g.*, if patient studied up to the third grade of Primary Education, tick "Primary Education".

[2] Consider "physical activity" sports practices such as walking, running, swimming and others. Disregard household activities such as brooming, washing, etc.

[3] Consider alcohol consumption in a single day and check patient gender in answer. If patient consumes less than 30g (men) or 15g (women) every day, tick "no". ". If consumption exceeds amounts indicated one or more days per week, tick "yes". Thirty grams of alcohol correspond to approximately two cans or bottles of beer, two glasses (150mL) of wine or two doses (50mL) of spirits (whisky, vodka and others).

8) Are you diabetic?

☐ Yes

☐ No

9) If your answered yes to question 8, how do you treat your diabetes problem?

☐ With diet

☐ With drugs

☐ No treatment was prescribed/do not treat

10) Do you have high blood pressure?

☐ Yes

☐ No

11) If your answered yes to question 10, how do you treat your high blood pressure problem?

☐ With diet

☐ With drugs

☐ No treatment was prescribed/do not treat

12) Have you ever had/do currently have:

☐ Infarction

☐ Stroke of CVA

☐ Kidney problems

☐ Visual problems[4]

13) In your family, has any of your relatives (up to first degree[5]) ever had: high blood pressure, high cholesterol, diabetes, infarction, stroke, CVA, renal problems or went blind?

☐ Yes

☐ No

14) Have you ever forgotten/chosen not to take any of the drugs prescribed to you by your physician?

☐ Yes

☐ No

15) Do you need help to take your medications?

☐ Yes

☐ No

16) Can you obtain all drugs prescribed to you by your physician here at the primary care unit pharmacy? If not, how do you get the remaining ones?[6]

☐ Yes

☐ No, I have, buy or am given some

☐ No, I cannot obtain some

17) Patient currently uses[7]:

☐ Drugs associated with HTN aggravation[8]

☐ Others[9]

[4] Do not consider myopia, hyperopia or astigmatism.

[5] Consider only parents and siblings.

[6] Check whether patient uses drugs other than those prescribed.

[7] Consider drugs prescribed other than those listed in prescriptions presented.

[8] Such as: immunosuppressants (cyclosporine and tacrolimus), anorexigenic drugs/satiety agents (amfepramone and others), anti-neoplastic agents (bevacizumab, estramustine, gemtuzumab ozogamicin and pazopanib), antidepressants (tricyclic and monoamine oxidase inhibitors), chronic use of corticosteroids, non-steroidal anti-inflammatory drugs, cyclooxygenase 1 and cyclooxygenase 2 inhibitors.

[9] Disregard antidyslipidemic, antidiabetic and antihypertensive drugs included in questions 7, 9 and 11, respectively

continue...

APPENDIX 2

Final Version of INSAF-HAS

Name:____

Address:____

Telephone: (__)_________ Mobile: (__)________ Date: _____/_____/_______

Record No.:__________________________ Date of birth:_____ _/____/_____

1) What is your age and sex (gender)*?

[1] Female, 49 years or less

[5] Female, 50 to 64 years

[9] Female, 65 years or older

[5] Male, 54 years or less

[9] Male, 55 years or older

2) What is your level of education (up to what grade did you study)*[1]?

[8] Illiterate

[4] Primary education (1st stage of education)

[0] Middle school (2nd stage or high school) or higher education

3) Do you practice physical exercise? (If yes, consider only frequency above or equal to that indicated in answer)*[2]:

[0] Yes, three times per week or more, for at least 30 minutes (90 minutes/week)

[6] No

4) Do you consume alcoholic drinks? If yes, how much?*[3]

a) If female patient:

[5] Yes, more than 15g in a single day

[0] No

b) If male patient:

[5] Yes, more than 30g in a single day

[0] No

5) Have you smoked/do you currently smoke cigarettes?*

[13] Yes

[5] Former smoker

[0] No

6) Have you ever forgotten/chosen not to take any of the drugs prescribed to you by your physician?*

[9] Yes

[0] No

7) Do you need help to take your medications?*

[7] Yes

[0] No

* Do not verbalize responses.

** Verbalize responses.

[1] Regardless of completion of a given stage, *e.g.*, if patient studied up to the third grade of Primary Education, tick "Primary Education".

[2] Consider "physical activity" sports practices such as walking, running, swimming and others. Disregard household activities such as brooming, washing, etc.

[3] Consider alcohol consumption in a single day and check patient gender in answer. If patient consumes less than 30g (men) or 15g (women) every day, tick "no". "If consumption exceeds amounts indicated one or more days per week, tick "yes". Thirty grams of alcohol correspond to approximately two cans or bottles of beer, two glasses (150mL) of wine or two doses (50mL) of spirits (whisky, vodka and others).

8) May I see your prescriptions? Do you take drugs other than those listed in these prescriptions? If yes, which? Analyze prescriptions and patient responses, then tick drugs used *[4]:

[7] Drugs potentially associated with HTN aggravation[5]

[4] Others[6]

9) Can you obtain all drugs prescribed to you by your physician here at the primary care unit pharmacy? If not, how do you get the remaining ones?*

[0] Yes

[3] No, I buy or am given some

[9] No, I cannot obtain some

continue...

...Continuation

APPENDIX 2

10) How do you treat your high blood pressure problem?**[7]

[8] With diet

[12] With drugs

[23] No treatment was prescribed/do not treat

11) Do you have high cholesterol?*[7]

[7] Yes

[0] No

12) If you answered yes to question 11, how do you treat your high cholesterol problem?** [7]

[1] With diet

[5] With drugs

[16] No treatment was prescribed/do not treat

13) Are you diabetic?*[7]

[7] Yes

[0] No

14) If you answered yes to question 13, how do you treat your diabetes problem?**[7]

[1] With diet

[5] With drugs

[16] No treatment was prescribed/do not treat

15) Have you ever had:**

[26] Infarction

[26] Stroke or CVA

16) In your family, has any of your first degree relatives (parents, siblings) ever had: high blood pressure, high cholesterol, diabetes, infarction, stroke, CVA?*

[2] Yes

[0] No

* Do not verbalize responses.

** Verbalize responses.

[4] Consider drugs prescribed other than those listed in prescriptions presented.

[5] Such as: immunosuppressants (cyclosporine and tacrolimus), anorexigenic drugs/satiety agents (amfepramone and others), anti-neoplastic agents (bevacizumab, estramustine, gemtuzumab ozogamicin and pazopanib), antidepressants (tricyclic – amitriptyline, nortriptyline, clomipramine, imipramine, maprotiline — and monoaminoxidase inhibitors - moclobemide and selegiline). Aside from these, consider chronic use of corticosteroids, non-steroidal anti-inflammatory drugs, cyclooxygenase 1 and cyclooxygenase 2 inhibitors. Acetylsalicylic acid may aggravate HTN when chronically used at doses exceeding 100mg.

[6] Disregard antidyslipidemic, antidiabetic and antihypertensive drugs included in questions 10, 12 and 14, respectively.

[7] Check whether responses to these questions reflect drugs prescribed. If patient declares not to suffer from one of these diseases but to use related drugs, select answer according to prescriptions presented.
