# Supplementary material for: INSAF-HAS: a tool to select patients with hypertension for pharmaceutical care
Source: Einstein (Sao Paulo). 2019 Dec 6;18:eAO4858. doi: 10.31744/einstein_journal/2020AO4858 (PMC6905168; doi:10.31744/einstein_journal/2020AO4858)
Supplement: Supplementary file 2 [file 2317-6385-eins-18-eAO4858-Suppl01-pt.pdf]

APÊNDICE 1

Versão inicial do INSAF-HAS

Nome:____

Nº do prontuário:________ Sexo:________ Data de nascimento: ___/___/____ Idade ________

1) Fatores de risco e doenças associadas

Pressão arterial (média) [X] mmHg

IMC (peso/altura^2):

Valor [ ]

- ☐ Obeso (grau______)
- ☐ Sobrepeso
- ☐ Normal

Fumante (marcar com x) *:

- ☐ Sim
- ☐ Não
- ☐ Ex-fumante

Dislipidemia (colesterol alto)*:

- ☐ Sim
- ☐ Não

Consumo de bebida alcoólica*:

- ☐ Sim (frequência__________)
- ☐ Não

Diabético*:

- ☐ Sim
- ☐ Não

Atividade física*:

- ☐ Sim (frequência__________)
- ☐ Não

2) História familiar (marcar com x, até segundo grau)*

- ☐ Hipertensão
- ☐ Dislipidemia
- ☐ Diabetes
- ☐ Perda visual**
- ☐ Doença cardiovascular
- ☐ Acidente vascular cerebral
- ☐ Insuficiência renal

3) Complicações (marcar com x) *

- ☐ Doença cardiovascular
- ☐ Acidente vascular cerebral
- ☐ Insuficiência renal
- ☐ Perda visual**

4) Número de medicamentos utilizados:

Total		Anti-hipertensivo	

* Relato do paciente
** Devido à hipertensão

Segunda versão do INSAF-HAS

Nome:____

Endereço:____

Telefone: (__)___________________ Celular: (__)__________________

Nº do prontuário:_________________ Data de nascimento:____ /______/______

1) Idade e gênero:

- ☐ Feminino, 65 anos ou menos
- ☐ Feminino, mais de 65 anos
- ☐ Masculino, 55 anos ou menos
- ☐ Masculino, mais de 55 anos

Marcar com 'x' a resposta dada pelo paciente às questões abaixo:

continua...

...Continuação

APÊNDICE 1

2) Você pratica atividade física? (Para a resposta sim, considerar apenas a frequência indicada na resposta)[1]:

☐ Sim, três ou mais vezes por semana, por no mínimo 30 minutos (90 minutos/semana)

☐ Não

3) Você consome bebida alcoólica?[2]:

Mulher:	Homem:
☐ Sim, mais de 15g em um mesmo dia	☐ Sim, mais de 30g em um mesmo dia
☐ Não	☐ Não

4) Você é tabagista (fumante):

☐ Sim

☐ Ex-fumante

☐ Não

5) Você tem colesterol alto:

☐ Sim

☐ Não

6) Você é diabético:

☐ Sim

☐ Não

7) Você tem pressão alta:

☐ Sim

☐ Não

[1] Considerar "atividade física" práticas esportivas como caminhada, corrida, natação e outros. Não considerar atividade do cuidado da casa como varrer, lavar roupas etc.

[2] Considerar o consumo de álcool em 1 único dia. Se o paciente consumir menos que 30g (no caso de homens) ou 15g (no caso de mulheres) todos os dias, assinalar a alternativa "não". Um ou mais dias da semana em que o consumo é superior ao indicado, assinalar a resposta "sim". Trinta gramas de álcool correspondem a, aproximadamente, duas latas ou uma garrafa de cerveja, duas taças (150mL) de vinho e duas doses (50mL) de destilados (uísque, vodca e aguardente).

8) Você já teve ou tem:

☐ Infarto

☐ Derrame ou AVC

☐ Problema renal

☐ Problema de visão[3]

9) Na sua família, entre os parentes de até segundo grau, alguém tem ou teve:

☐ Pressão alta

☐ Colesterol alto

☐ Diabetes

☐ Ficou cego

☐ Infarto

☐ Derrame ou AVC

☐ Problema renal

10) Você alguma vez esquece ou deixa de tomar algum medicamento receitado pelo médico:

☐ Sim ☐ Não

11) Você consegue pegar todos os medicamentos prescritos pelo médico aqui na farmácia do posto de saúde? Se não, como você obtêm os outros?[4]

☐ Sim

☐ Não, algum(s) eu tenho, compro ou ganho

☐ Não, algum(s) eu não tenho como obter

12) Número de medicamentos utilizados[5]:

☐ Medicamentos para pressão

☐ Medicamentos para diabetes

☐ Medicamentos para colesterol

☐ Medicamentos relacionados ao agravamento da HAS[6]

☐ Outros

☐ Total

continua...

...Continuação

APÊNDICE 1

[3] Não considerar miopia, hipermetropia e astigmatismo e outros que não sejam secundários à hipertensão arterial sistêmica.

[4] Verificar se o paciente utiliza outros medicamentos, além dos prescritos na receita.

[5] Considerar também outros medicamentos prescritos, mas que não estão nas receitas apresentadas.

[6] São eles: imunossupressores (ciclosporina e tacrolimus), anorexígenos/sacietógenos (anfepramona e outros), antineoplásicos (bevacizumabe, estramustina, gemtuzumabe ozogamicina e pazopanibe), antidepressivos (tricíclicos e inibidores da monoaminoxidase), uso crônico de corticoides, anti-inflamatórios não esteroidais, inibidores da ciclo-oxigenase 1 e ciclo-oxigenase 2.

Terceira versão do INSAF-HAS

Nome:____

Endereço:____

Telefone: (___)____________________ Celular: (___)___________________ Data: _____/_____/_______

Nº do prontuário:______________ Data Nascimento:________/________/________

1) Qual a sua idade e seu gênero (sexo)?

☐ Feminino, 49 anos ou menos

☐ Feminino, 50 a 65 anos

☐ Feminino, mais de 65 anos

☐ Masculino, 55 anos ou menos

☐ Masculino, mais de 55 anos

2) Qual a sua escolaridade (quanto você estudou)?

☐ Analfabeto

☐ Fundamental incompleto

☐ Fundamental completo

☐ Médio incompleto

☐ Médio completo

☐ Superior incompleto

☐ Superior completo

3) Você pratica exercício físico? (Para a resposta sim, considerar apenas a frequência igual ao superior a indicada na resposta)[1]:

☐ Sim, três ou mais vezes por semana, por no mínimo 30 minutos (90 minutos/semana)

☐ Não

4) Você consome bebida alcoólica?[2]

a) Se paciente do gênero feminino:

☐ Sim, mais de 15g em um mesmo dia

☐ Não

b) Se paciente do gênero masculino:

☐ Sim, mais de 30g em um mesmo dia

☐ Não

5) Você é tabagista (fumante)?

☐ Sim

☐ Ex-fumante

☐ Não

6) Você tem colesterol alto?

☐ Sim

☐ Não

7) Você é diabético?

☐ Sim

☐ Não

8) Você tem pressão alta?

☐ Sim

☐ Não

[1] Considerar "exercício físico" práticas esportivas como caminhada, corrida, natação e outros. Não considerar atividade do cuidado doméstico, como varrer, lavar roupas etc.

[2] Considerar o consumo de álcool em 1 único dia e observar o gênero do paciente na resposta. Se o paciente consumir menos que 30g (no caso de homens) ou 15g (no caso de mulheres) todos os dias, assinalar a alternativa "não". Um ou mais dias da semana em que o consumo é superior ao indicado, assinalar a resposta "sim". Trinta gramas de álcool correspondem a, aproximadamente, duas latas ou uma garrafa de cerveja, duas taças (150mL) de vinho e duas doses (50mL) de destilados (uísque, vodca e aguardente).

continua...

...Continuação

APÊNDICE 1

9) Você já teve ou tem:

☐ Infarto

☐ Derrame ou AVC

☐ Problema renal

☐ Problema de visão[3]

10) Na sua família, entre os parentes de até segundo grau, alguém tem ou teve:

☐ Pressão alta

☐ Colesterol alto

☐ Diabetes

☐ Ficou cego

☐ Infarto

☐ Derrame ou AVC

☐ Problema renal

11) Você alguma vez esquece ou deixa de tomar algum medicamento receitado pelo médico?

☐ Sim

☐ Não

12) Você precisa de ajuda para tomar os seus medicamentos?

☐ Sim

☐ Não

13) Você consegue pegar todos os medicamentos prescritos pelo médico aqui na farmácia do posto de saúde? Se não, como você obtêm os outros?[4]

☐ Sim

☐ Não, algum(s) eu tenho, compro ou ganho.

☐ Não, algum(s) eu não tenho como obter

14) O paciente utiliza[5]:

☐ Medicamentos para pressão

☐ Medicamentos para diabetes

☐ Medicamentos para colesterol

☐ Medicamentos relacionados ao agravamento da HAS[6]

☐ Outros

[3] Não considerar miopia, hipermetropia e astigmatismo.

[4] Verificar se o paciente utiliza outros medicamentos além dos prescritos na receita.

[5] Considerar também outros medicamentos prescritos, mas que não estão nas receitas apresentadas. Marcar apenas as classes de medicamentos utilizadas pelo paciente e descritas na resposta da questão.

[6] São eles: imunossupressores (ciclosporina e tacrolimus), anorexígenos/sacietógenos (anfepramona e outros), antineoplásicos (bevacizumebe, estramustina, gemtuzumabe ozogamicina e pazopanibe), antidepressivos (tricíclicos e inibidores da monoaminoxidase), uso crônico de corticoides, anti-inflamatórios não esteroidais, inibidores da ciclo-oxigenase 1 e ciclo-oxigenase 2.

Quarta versão do INSAF-HAS

Nome:____

Endereço:____

Telefone: (___)____________________ Celular: (___)___________________ Data: _____/_____/_______

Nº do prontuário:______________ Data Nascimento:________/________/________

1) Qual a sua idade e seu gênero (sexo)?

☐ Feminino, 49 anos ou menos

☐ Feminino, 50 a 65 anos

☐ Feminino, mais de 65 anos

☐ Masculino, 55 anos ou menos

☐ Masculino, mais de 55 anos

2) Qual a sua escolaridade (quanto você estudou)?

☐ Analfabeto

☐ Até Ensino Fundamental completo

☐ Até Ensino Médio completo

☐ Até Ensino Superior completo

continua...

...Continuação

APÊNDICE 1

3) Você pratica exercício físico? (para a resposta sim, considerar apenas a frequência igual ao superior a indicada na resposta)[1]:

- ☐ Sim, três ou mais vezes por semana, por no mínimo 30 minutos (90 minutos/semana)
- ☐ Não

4) Você consome bebida alcoólica?[2]

a) Se paciente do gênero feminino:	b) Se paciente do gênero masculino:
☐ Sim, mais de 15g em um mesmo dia	☐ Sim, mais de 30g em um mesmo dia
☐ Não	☐ Não

5) Você é tabagista (fumante)?

- ☐ Sim
- ☐ Ex-fumante
- ☐ Não

6) Você tem colesterol alto?

- ☐ Sim
- ☐ Não

7) Se você respondeu sim para a pergunta 6, como você trata seu problema de colesterol alto?

- ☐ Com dieta
- ☐ Com medicamentos
- ☐ O médico não indicou tratamento/não trato

8) Você é diabético?

- ☐ Sim
- ☐ Não

[1] Considerar "exercício físico" práticas esportivas como caminhada, corrida, natação e outros. Não considerar atividade do cuidado doméstico como varrer, lavar roupas etc.

[2] Considerar o consumo de álcool em 1 único dia e observar o gênero do paciente na resposta. Se o paciente consumir menos que 30g (no caso de homens) ou 15g (no caso de mulheres) todos os dias, assinalar a alternativa "não". Um ou mais dias da semana em que o consumo é superior ao indicado, assinalar a resposta "sim". Trinta gramas de álcool correspondem a, aproximadamente, duas latas ou uma garrafa de cerveja, duas taças (150mL) de vinho e duas doses (50mL) de destilados (uísque, vodca e aguardente).

9) Se você respondeu sim para a pergunta 8, como você trata seu problema de diabetes?

- ☐ Com dieta
- ☐ Com medicamentos
- ☐ O médico não indicou tratamento/não trato

10) Você tem pressão alta?

- ☐ Sim
- ☐ Não

11) Se você respondeu sim para a pergunta 10, como você trata seu problema de pressão alta?

- ☐ Com dieta
- ☐ Com medicamentos
- ☐ O médico não indicou tratamento/ não trato

12) Você já teve ou tem:

- ☐ Infarto
- ☐ Derrame ou AVC
- ☐ Problema renal
- ☐ Problema de visão[3]

13) Na sua família, entre os parentes de até primeiro grau[4], alguém tem ou teve:

- ☐ Pressão alta
- ☐ Colesterol alto
- ☐ Diabetes
- ☐ Ficou cego
- ☐ Infarto
- ☐ Derrame ou AVC
- ☐ Problema renal

continua...

...Continuação

APÊNDICE 1

14) Você alguma vez esquece ou deixa de tomar algum medicamento receitado pelo médico?

☐ Sim

☐ Não

15) Você precisa de ajuda para tomar os seus medicamentos?

☐ Sim

☐ Não

16) Você consegue pegar todos os medicamentos prescritos pelo médico aqui na farmácia do posto de saúde? Se não, como você obtêm os outros?[5]

☐ Sim

☐ Não, algum(s) eu tenho, compro ou ganho

☐ Não, algum(s) eu não tenho como obter

17) O paciente utiliza[6]:

☐ Medicamentos relacionados ao agravamento da HAS[7]

☐ Outros[8]

[3] Não considerar miopia, hipermetropia e astigmatismo.

[4] Considerar apenas pais e irmãos.

[5] Verificar se o paciente utiliza outros medicamentos além dos prescritos na receita.

[6] Considerar também outros medicamentos prescritos, mas que não estão nas receitas apresentadas.

[7] São eles: imunossupressores (ciclosporina e tacrolimus), anorexígenos/sacietógenos (anfepramona e outros), antineoplásicos (bevacizumebe, estramustina, gemtuzumabe ozogamicina e pazopanibe), antidepressivos (tricíclicos e inibidores da monoaminoxidase), uso crônico de corticoides, anti-inflamatórios não esteroidais, inibidores da ciclo-oxigenase 1 e ciclo-oxigenase 2.

[8] Desconsiderar também medicamentos para dislipidemia, diabetes e hipertensão, já contemplados nas questões 7, 9 e 11, respectivamente.

Quinta versão do INSAF-HAS

Nome:____

Endereço:____

Telefone: (___)____________Celular: (___)________Data: _____/_____/_______

Nº do prontuário:______________ Data de nascimento:______ /_______/________

1) Qual a sua idade e seu gênero (sexo)?

☐ Feminino, 49 anos ou menos

☐ Feminino, 50 a 64 anos

☐ Feminino, 65 anos ou mais

☐ Masculino, 54 anos ou menos

☐ Masculino, 55 anos ou mais

2) Qual a sua escolaridade (quanto você estudou)[1]?

☐ Analfabeto

☐ Ensino Fundamental (1º grau ou ginásio)

☐ Ensino Médio (2º grau ou colegial) ou superior

3) Você pratica exercício físico? (Para a resposta sim, considerar apenas a frequência igual ao superior à indicada na resposta)[2]:

☐ Sim, três ou mais vezes por semana, por no mínimo 30 minutos (90 minutos/semana)

☐ Não

4) Você consome bebida alcoólica?[3]

a) Se paciente do gênero feminino:

☐ Sim, mais de 15g em um mesmo dia

☐ Não

b) Se paciente do gênero masculino:

☐ Sim, mais de 30g em um mesmo dia

☐ Não

5) Você é tabagista (fumante)?

☐ Sim

☐ Ex-fumante

☐ Não

6) Você tem colesterol alto?

☐ Sim

☐ Não

continua...

...Continuação

APÊNDICE 1

7) Se você respondeu sim para a pergunta 6, como você trata seu problema de colesterol alto?

☐ Com dieta

☐ Com remédios

☐ O médico não indicou tratamento/ não trato

[1] Independente do paciente ter ou não completado determinada fase. Por exemplo, se o paciente estudou até a terceira série do Ensino Fundamental, marcar a opção "Ensino Fundamental".

[2] Considerar "exercício físico" práticas esportivas como caminhada, corrida, natação e outros. Não considerar atividade do cuidado doméstico como varrer, lavar roupas etc.

[3] Considerar o consumo de álcool em 1 único dia e observar o gênero do paciente na resposta. Se o paciente consumir menos que 30g (no caso de homens) ou 15g (no caso de mulheres) todos os dias, assinalar a alternativa "não". Um ou mais dias da semana em que o consumo é superior ao indicado, assinalar a resposta "sim". Trinta gramas de álcool correspondem a, aproximadamente, duas latas ou uma garrafa de cerveja, duas taças (150mL) de vinho e duas doses (50mL) de destilados (uísque, vodca e aguardente).

8) Você é diabético?

☐ Sim

☐ Não

9) Se você respondeu sim para a pergunta 8, como você trata seu problema de diabetes?

☐ Com dieta

☐ Com remédios

☐ O médico não indicou tratamento/não trato

10) Você tem pressão alta?

☐ Sim

☐ Não

11) Se você respondeu sim para a pergunta 10, como você trata seu problema de pressão alta?

☐ Com dieta

☐ Com remédios

☐ O médico não indicou tratamento/ não trato

12) Você já teve ou tem:

☐ Infarto

☐ Derrame ou AVC

☐ Problema renal

☐ Problema de visão[4]

13) Na sua família, entre os parentes de até primeiro grau[5], alguém tem ou teve: pressão alta, colesterol alto, diabetes, infarto, derrame, AVC, problema renal ou ficou cego?

☐ Sim

☐ Não

14) Você alguma vez esquece ou deixa de tomar algum remédio receitado pelo médico?

☐ Sim

☐ Não

15) Você precisa de ajuda para tomar os seus remédios?

☐ Sim

☐ Não

16) Você consegue pegar todos os remédios receitados pelo médico aqui na farmácia do posto de saúde? Se não, como você obtêm?[6]

☐ Sim

☐ Não, algum(s) eu compro ou ganho

☐ Não, algum(s) eu não tenho como obter

17) O paciente utiliza[7]:

☐ Medicamentos relacionados ao agravamento da HAS[8]

☐ Outros[9]

[4] Não considerar miopia, hipermetropia e astigmatismo.

[5] Considerar apenas pais e irmãos.

[6] Verificar se o paciente utiliza outros medicamentos além dos prescritos na receita.

[7] Considerar também outros medicamentos prescritos, mas que não estão nas receitas apresentadas.

[8] São eles: imunossupressores (ciclosporina e tacrolimus), anorexígenos/sacietógenos (anfepramona e outros), antineoplásicos (bevacizumebe, estramustina, gemtuzumabe ozogamicina e pazopanibe), antidepressivos (tricíclicos e inibidores da monoaminoxidase), uso crônico de corticoides, anti-inflamatórios não esteroidais, inibidores da ciclo-oxigenase 1 e ciclo-oxigenase 2.

[9] Desconsiderar medicamentos para dislipidemia, diabetes e hipertensão, já contemplados nas questões 7, 9 e 11, respectivamente.

APÊNDICE 2

Versão final do INSAF-HAS

Nome:____

Endereço:____

Telefone: (___)____________Celular: (___)_________Data: _____/_____/_______

Nº do prontuário:___________________ Data de nascimento:_____ _/____/_____

1) Qual a sua idade e seu sexo (gênero)*?

[1] Feminino, 49 anos ou menos

[5] Feminino, 50 a 64 anos

[9] Feminino, 65 anos ou mais

[5] Masculino, 54 anos ou menos

[9] Masculino, 55 anos ou mais

2) Qual a sua escolaridade (até que ano você estudou)*[1]?

[8] Analfabeto

[4] Ensino Fundamental (1º grau ou ginásio)

[0] Ensino Médio (2º grau ou colegial) ou superior

3) Você pratica exercício físico? Se sim, quanto? Com que frequência? (Para a resposta sim, considerar apenas a frequência igual ao superior a indicada na resposta)*[2]:

[0] Sim, três ou mais vezes por semana, por, no mínimo, 30 minutos (90 minutos/semana)

[6] Não

4) Você consome bebida alcoólica? Se sim, quanto?*[3]

a) Se paciente do gênero feminino:

[5] Sim, mais de 15g em um mesmo dia

[0] Não

b) Se paciente do gênero masculino:

[5] Sim, mais de 30g em um mesmo dia

[0] Não

5) Você foi ou é tabagista (fumante)?*

[13] Sim [5] Ex-fumante [0] Não

6) Você alguma vez esquece ou deixa de tomar algum remédio receitado pelo médico?*

[9] Sim [0] Não

7) Você precisa de ajuda para tomar os seus remédios?*

[7] Sim [0] Não

* Não verbalizar as respostas.

** Verbalizar as respostas.

[1] Independente do paciente ter ou não completado determinada etapa. Por exemplo, se o paciente estudou até a terceira série do Ensino Fundamental, marcar a opção "Ensino Fundamental".

[2] Considerar como "exercício físico" práticas esportivas como caminhada, corrida, natação e outros. Não considerar atividade do cuidado doméstico como varrer, lavar roupas etc.

[3] Considerar o consumo de álcool em 1 único dia e observar o gênero do paciente na resposta. Se o paciente consumir menos que 30g (no caso de homens) ou 15g (no caso de mulheres) todos os dias, assinalar a alternativa "não". Um ou mais dias da semana em que o consumo é superior ao indicado, assinalar a resposta "sim". Trinta gramas de álcool correspondem a, aproximadamente, duas latas ou uma garrafa de cerveja, duas taças (150mL) de vinho e duas doses (50mL) de destilados (uísque, vodca e aguardente).

8) Eu posso ver suas receitas? Você utiliza outros medicamentos além destes nesta(s) receita(s)? Se sim, o que? Analisar as receitas e a resposta do paciente e marcar os medicamentos que o paciente utiliza*[4]:

[7] Medicamentos que podem agravar a HAS[5]

[4] Outros[6]

9) Você consegue pegar todos os remédios receitados pelo médico aqui na farmácia do posto de saúde? Se não, como você obtêm?*

[0] Sim

[3] Não, algum(s) eu compro ou ganho

[9] Não, algum(s) eu não tenho como obter

10) Como você trata seu problema de pressão alta?**[7]

[8] Com dieta

[12] Com remédios

[23] O médico não indicou tratamento/não trato

continua...

...Continuação

APÊNDICE 2

11) Você tem colesterol alto?*[7]

[7] Sim

[0] Não

12) Se sim para a pergunta 11, como você trata seu problema de colesterol alto?** [7]

[1] Com dieta

[5] Com remédios

[16] O médico não indicou tratamento/ não trato

13) Você é diabético?*[7]

[7] Sim

[0] Não

14) Se sim para a pergunta 13, como você trata seu problema de diabetes?** [7]

[1] Com dieta

[5] Com remédios

[16] O médico não indicou tratamento/ não trato

15) Você já teve:**

[26] Infarto

[26] Derrame ou AVC

16) Na sua família, entre os parentes de primeiro grau, pais e irmãos, alguém tem ou teve: pressão alta, colesterol alto, diabetes, infarto, derrame ou AVC?*

[2] Sim [0] Não

* Não verbalizar as respostas.

** Verbalizar as respostas.

[4] Considerar também outros medicamentos prescritos, mas que não estão nas receitas apresentadas.

[5] São eles: imunossupressores (ciclosporina e tacrolimus), anorexígenos/sacietógenos (anfepramona e outros), antineoplásicos (bevacizumebe, estramustina, gemtuzumabe ozogamicina e pazopanibe) e antidepressivos (tricíclicos – amitriptilina, nortriptilina, clomipramina, imipramina, maprotilina – e inibidores da monoaminoxidase – moclobemida e selegilina). Além desses, quando utilizados de forma crônica, considerar também os grupos de fármacos: corticoides, anti-inflamatórios não esteroidais, inibidores da ciclo-oxigenase 1 e ciclo-oxigenase 2. O ácido acetilsalicílico pode agravar a HAS quando utilizado em dose maior que 100 mg e de forma crônica.

[6] Desconsiderar medicamentos para dislipidemia, diabetes e hipertensão, que são contemplados nas questões 10, 12 e 14, respectivamente.

[7] Conferir se a resposta dessas perguntas coincide com os medicamentos prescritos. Caso o paciente afirme não ter uma dessas doenças, mas utilizar medicamento para ela, deve-se marcar a resposta com base nas prescrições apresentadas.
