# Supplementary material for: INSAF-HAS: a tool to select patients with hypertension for pharmaceutical care
Source: Einstein (Sao Paulo). 2019 Dec 6;18:eAO4858. doi: 10.31744/einstein_journal/2020AO4858 (PMC6905168; doi:10.31744/einstein_journal/2020AO4858)
Supplement: Translation

# Translation: INSAF-HAS: ferramenta para seleção de pacientes com hipertensão arterial sistêmica e inserção em serviços de cuidado farmacêutico

**Authors:** Beatriz Maria Pereira Girolineto, Alan Maicon de Oliveira, Ana Maria Rosa Freato Gonçalves, Marília Silveira de Almeida Campos, Leonardo Régis Leira Pereira

**Affiliations:** 1 Universidade Federal do Piauí, Teresina, PI, Brasil.; 2 Faculdade de Ciências Farmacêuticas de Ribeirão Preto, Universidade de São Paulo, Ribeirão Preto, SP, Brasil.

**Keywords:** Assistência farmacêutica, Hipertensão, Anti-hipertensivos, Inquéritos e questionários, Triagem, Relações profissional-paciente, Gestão de qualidade, Seleção de pacientes

## Abstract

**Objetivo:**

Desenvolver e realizar a validação de conteúdo de uma ferramenta para seleção de pacientes com hipertensão arterial sistêmica a serem atendidos em serviços de cuidado farmacêutico, com base na identificação daqueles com maior necessidade dessa assistência.

**Métodos:**

O instrumento foi desenvolvido e avaliado por validação de face e conteúdo, subdividida em três fases. A fase I abordou ampla revisão bibliográfica, que originou a versão inicial da ferramenta. Na fase II, realizou-se a validação com a análise de um painel de *experts* . A fase III foi composta por estudo piloto realizado com pacientes com hipertensão, tendo sido definida a versão final do instrumento.

**Resultados:**

A partir da revisão bibliográfica, 30 estudos foram consultados, e foram selecionados 13 fatores associados ao controle e à ocorrência de complicações, relacionados à hipertensão arterial sistêmica e a doenças cardiovasculares. Por conseguinte, mediante a versão inicial da ferramenta intitulada INSAF-HAS, realizaram-se quatro reuniões com especialistas, para obtenção de consenso final. A cada encontro, o instrumento foi aprimorado. No estudo piloto, 30 pacientes com diagnóstico de hipertensão arterial sistêmica foram abordados para o pré-teste de avaliação da aplicabilidade e, após as adequações, obteve-se a versão final do INSAF-HAS.

**Conclusão:**

A ferramenta elaborada INSAF-HAS apresenta validade de face e conteúdo. Ela deve contribuir para a seleção de pacientes portadores de hipertensão arterial sistêmica e com maior necessidade de participação em serviços de cuidado farmacêutico.

## INTRODUÇÃO

A hipertensão arterial sistêmica (HAS) é uma condição clínica multifatorial caracterizada por elevação sustentada dos níveis pressóricos, que pode levar à lesão de órgãos-alvo.^1^ Em 2010, a prevalência de hipertensão foi de 28,5% (27,3% a 29,7%) em países de renda elevada e 31,5% (30,2% a 32,9%) naqueles de renda baixa e média, como o Brasil.^2^ Pesquisas indicam que a prevalência de HAS na população brasileira varia entre 22,3% e 64,1%.^3 - 9^

A falta de controle da HAS pode levar ao desenvolvimento e/ou agravamento de doenças cardiovasculares, como o acidente vascular cerebral (AVC), infarto agudo do miocárdio (IAM) e insuficiência cardíaca.^10^ Assim, torna-se fundamental o tratamento da doença, a fim de evitar tais eventos.^10^

A farmacoterapia contribui para o manejo da HAS, porém existem evidências de que o controle pressórico de pacientes hipertensos está aquém do desejável, e entre 15,5% e 63% deles alcançam esse objetivo.^11 - 15^ Vários fatores podem contribuir para a não efetividade da farmacoterapia, como erros de medicação, prescrição inadequada e não adesão ao tratamento, entre outros.

A efetividade da atuação clínica do farmacêutico tem sido consistentemente demonstrada em estudos por meio da melhoria da qualidade de vida dos pacientes atendidos, da identificação e resolução de problemas farmacoterapêuticos, da adequação de parâmetros clínicos e da redução de gastos para doenças crônicas, como o *diabetes mellitus* (DM), a dislipidemia e a HAS.^16 - 21^ Isso evidencia que a inserção de serviços de cuidado farmacêutico^22^ na prestação de assistência à saúde constitui estratégia eficaz para promover impactos clínicos, humanísticos e econômicos.^23 - 30^

Entretanto, no Brasil, encontramos um quadro de profissionais farmacêuticos com grande demanda de atividades gerenciais, o que dificulta o acesso ao serviço clínico para os pacientes que necessitam de atendimento individualizado.^31 - 33^ A fim de ampliar essa abordagem e direcioná-la a pacientes com mais necessidades farmacoterapêuticas, faz-se necessário o desenvolvimento de ferramentas de triagem de pacientes, criticamente relacionadas ao sucesso da prática clínica farmacêutica.^34^

## OBJETIVO

Desenvolver e validar uma ferramenta de seleção de pacientes com hipertensão arterial sistêmica, com mais necessidades terapêuticas, para serem incluídos em serviços de cuidado farmacêutico.

## MÉTODOS

O estudo desenvolveu-se em três fases para desenvolvimento e validação de uma ferramenta de seleção de pacientes com HAS intitulada INSAF-HAS ( Figura 1 ). Na fase I, uma revisão bibliográfica foi realizada, para a elaboração da ferramenta em sua versão inicial. A fase II consistiu na avaliação do instrumento pelo painel de *experts* . Na fase III, realizou-se piloto para teste da compreensão das perguntas e praticidade de resposta, definindo-se a versão final da ferramenta. Este estudo foi aprovado pelo Comitê de Ética em Pesquisa da Faculdade de Ciências Farmacêuticas de Ribeirão Preto da Universidade de São Paulo por meio do ofício número 67/2011.

**Figura 1:** Processo de desenvolvimento da ferramenta, por meio da validação de face e conteúdo HAS: hipertensão arterial sistêmica.

### Fase I: revisão bibliográfica e desenvolvimento do instrumento

Inicialmente, uma revisão bibliográfica foi realizada para identificar os fatores que podem determinar os resultados clínicos relacionados à HAS. Para isso, realizou-se busca na base de dados PubMed^®^com os termos *Medical Subject Headings* (MeSH) relacionados ao cuidado farmacêutico ( *pharmaceutical services* ), à HAS ( *hypertension* e *risk factors* ) e à adesão ao tratamento medicamentoso ( *medication adherence* e *patient compliance* ).

Após a seleção dos artigos, verificaram-se também outros textos referenciados nos primeiros estudos e que se associavam ao assunto. Com base em leitura criteriosa dos documentos, os autores elaboraram a versão inicial da mesma ferramenta INSAF-HAS.

### Fase II: painel de *experts*

A primeira versão foi submetida à avaliação de cinco especialistas, que eram profissionais farmacêuticos com experiência consolidada (ensino, pesquisa e prática profissional) em serviços de cuidado farmacêutico e HAS, todos doutorandos na área.^35^ Instituiu-se o painel de *experts* em reuniões presenciais para análise da pertinência e clareza de cada item incluído. Além disso, os especialistas tiveram oportunidade de refinar os conceitos e sugerir a inclusão de elementos que, de acordo com suas experiências, estão associados ao controle ou não da HAS.

### Fase III: estudo piloto e definição do instrumento oficial

Em seguida, realizou-se um estudo piloto, a fim de identificar as possíveis dificuldades de entendimento, e a praticidade de resposta e de aplicação da ferramenta. Essa etapa ocorreu em uma unidade de Atenção Básica à Saúde do município de Ribeirão Preto (SP), Brasil. Um farmacêutico devidamente capacitado, com a finalidade de minimizar vieses no preenchimento e/ou na indução de respostas, aplicou o instrumento (face a face) em uma amostra de conveniência de pacientes com diagnóstico de HAS, com idade igual ou superior a 18 anos e em uso de ao menos um medicamento anti-hipertensivo. O recrutamento foi voluntário e aconteceu quando os indivíduos foram à farmácia do referido estabelecimento de saúde para a retirada de seus medicamentos para hipertensão.

As dificuldades constatadas foram discutidas e, por meio de consensos do painel de especialistas, modificaram-se a redação e os aspectos pertinentes. O estudo piloto terminou no momento em que 11 participantes consecutivos relataram e demonstraram compreensão de todos os itens do instrumento.

Após este estudo, obteve-se a versão final e, de acordo com avaliação realizada pelos especialistas, cada item recebeu um peso (pontuação) que compôs o escore final da ferramenta.

## RESULTADOS

### Fase I

A partir da revisão bibliográfica, 30 estudos (incluem-se diretrizes) foram consultados para a definição dos indicadores a serem inseridos no instrumento. Após a revisão crítica, foram selecionados os fatores que a literatura apontou como associados ao controle da pressão arterial (PA), na ocorrência de complicações e agravamentos relacionados à HAS e de doenças cardiovasculares, bem como na adesão ao tratamento farmacológico prescrito e na ocorrência de problemas relacionados à farmacoterapia ( Tabela 1 ). Entretanto, os fatores cujo efeito ainda é controverso, como estresse, depressão e consumo de café, não foram incluídos.

**Tabela 1:** Fatores determinantes em resultados clínicos relacionados à progressão da hipertensão arterial sistêmica

Fatores	Estudos
Idade e sexo	Sociedade Brasileira de Cardiologia^(36)^
	Gueyffier et al.^(37)^
	Takahashi et al.^(38)^
Escolaridade	Vasconcelos et al.^(39)^
Prática regular de exercícios físicos	Sociedade Brasileira de Cardiologia^(36)^
	O´Donnell et al.^(40)^
	Pescatello et al.^(41)^
Tabagismo	Sociedade Brasileira de Cardiologia^(36)^
	Gueyffier et al.^(37)^
	O´Donnell et al.^(40)^
	Ridker et al.^(42)^
	Rempher^(43)^
Etilismo	Sociedade Brasileira de Cardiologia^(36)^
	Parekh et al.^(44)^
	Taylor et al.^(45)^
Adesão à farmacoterapia	Vasconcelos et al.^(39)^
	Bunker et al.^(46)^
	Balkrishnan^(47)^
Acesso aos medicamentos	Vasconcelos et al.^(39)^Balkrishnan^(47)^
Necessidade de cuidador	Vasconcelos et al.^(39)^
Uso de medicamentos que podem aumentar a PA	Sociedade Brasileira de Cardiologia^(36)^
	Amer et al.^(48)^
	Snowden et al.^(49)^
Comorbidades: *diabetes mellitus* e dislipidemia	Gueyffier et al.^(37)^
	Ridker et al.^(42)^
	Piegas et al.^(50)^
	Cubeddu et al.^(51)^
	Reaven.^(52)^
Ocorrência prévia de agravamentos: IAM e AVC	Gueyffier et al.^(37)^
	Schmieder^(53)^
Histórico familiar: HAS, dislipidemia, *diabetes mellitus,* IAM e AVC	Harrison et al.^(54)^
	Kennedy et al.^(55)^

PA: pressão arterial; IAM: infarto agudo do miocárdio; AVC: acidente vascular cerebral; HAS: hipertensão arterial sistêmica.

Obteve-se, então, a versão inicial do INSAF-HAS ( Apêndice 1 ). No decorrer das avaliações, os fatores cuja mensuração poderia afetar a praticidade do uso do instrumento e a fidedignidade das respostas (obesidade, ingestão de sal e a manifestação prévia de insuficiência renal ou retinopatia hipertensiva) também foram excluídos.

### Fase II

Foram realizadas quatro reuniões de análise e discussão com os especialistas, até obtenção de consenso final sobre a ferramenta ( Figura 2 ). Durante as reuniões, avaliaram-se a clareza dos itens e a relevância de cada indicador, sendo designada a pontuação para cada um deles e, consequentemente, a pontuação final. A cada reunião, a ferramenta foi modificada (versões 2 a 5) ( Apêndice 1 ).

**Figura 2:** Fluxograma de desenvolvimento do INSAF-HAS

De acordo com a importância do indicador na evolução clínica da HAS, pesos diferentes foram atribuídos para os distintos fatores. Assim, para o INSAF-HAS, desenvolveu-se um escore que variava de 9 a 200 pontos ( Tabela 2 ); quanto menor a pontuação, menor a prioridade do indivíduo em receber o cuidado farmacêutico nesse contexto.

**Tabela 2:** Pontuação mínima e máxima para cada questão do INSAF-HAS

Questão do formulário	Pontuação mínima	Pontuação máxima
1	1	9
2	0	8
3	0	6
4	0	5
5	0	13
6	0	9
7	0	7
8	0	11
9	0	9
10	8	23
11	0	7
12	0	16
13	0	7
14	0	16
15	0	52
16	0	2
**Total**	9	200

### Fase III

Após consenso final do painel de *experts* sobre o INSAF-HAS, iniciou-se o pré-teste, para verificar a aplicabilidade do mesmo, ou seja, se os pacientes compreendiam ou não as perguntas. Ao todo, 30 pacientes com diagnóstico de HAS foram abordados no momento da dispensação de medicamentos na Unidade Básica de Saúde, até que 11 participantes consecutivos demonstraram entendimento adequado de todos os itens do instrumento.

Durante esse período, o formulário passou por novas modificações ( Tabela 3 ). Algumas questões foram reescritas para melhorar a interpretabilidade dos pacientes e compuseram a versão final do INSAF-HAS ( Apêndice 2 ).

**Tabela 3:** Modificações realizadas no INSAF-HAS após o estudo piloto

Versão 5 do INSAF-HAS	Motivo da alteração	Versão final do INSAF-HAS
Qual a sua escolaridade (quanto você estudou)?	Dificuldades de entendimento no discurso	Qual a sua escolaridade (até que ano você estudou)?
Você consome bebida alcoólica?	Pergunta mais clara para auxiliar o entrevistador	Você consome bebida alcoólica? Se sim, quanto?
Os itens 6 a 9, sobre comorbidades	A ordem dos itens foi alterada para se adequar à análise da prescrição medicamentosa	Nova ordem: itens 11 a 14
Você tem pressão alta?	Muitos pacientes não sabiam responder sobre o controle pressórico, e isso gerou dúvida, uma vez que todos tinham diagnóstico de HAS	Retirada
Se você respondeu “sim” para a pergunta 10, como você trata seu problema de pressão alta?	Alteração devido à retirada do item 10	Como você trata seu problema de pressão alta?
Os itens 12 e 13, sobre agravamentos da HAS e histórico familiar, respectivamente	A ordem dos itens foi alterada para adequar à análise da prescrição medicamentosa e dinâmica da entrevista com o paciente	Nova ordem: itens 15 e 16
Os itens 14, 15 e 16, sobre adesão e acesso aos medicamentos	A ordem dos itens foi alterada para adequar à análise da prescrição medicamentosa e à dinâmica da entrevista com o paciente	Nova ordem: itens 6, 7 e 9, respectivamente

## DISCUSSÃO

O presente estudo resultou no desenvolvimento de uma ferramenta diferenciada para analisar fatores relacionados à terapêutica e também aspectos relativos ao quadro clínico do indivíduo com HAS. Existem poucas evidências científicas que disponibilizam instrumentos de triagem para usuários de serviços de cuidado farmacêutico, e a maioria dos estudos considera, de maneira distinta, os problemas relacionados à farmacoterapia e às condições clínicas do paciente, sem avaliar o perfil do indivíduo de forma holística.^56 - 60^ É importante ressaltar que o INSAF-HAS não é uma ferramenta de inclusão ou exclusão, mas um instrumento que indica a prioridade de atendimento dos indivíduos. À vista disso, após o alcance de resultados significativos dos pacientes selecionados, novos poderão atender aos critérios de prioridade e receber a assistência individual com base no cuidado farmacêutico.

As novas demandas dos sistemas de saúde que surgem no momento atual destacam a importância dos serviços clínicos e de educação em saúde pública disponibilizados pelo farmacêutico.^61 , 62^ Apesar disso, revela-se a escassez de aspectos motivadores, para que esse profissional possa aplicar os conhecimentos em suas práticas, considerando-se o perfil tecnicista predominante ainda em diversos serviços (principalmente em países de baixa e média renda). Há também superlotação por conta da busca elevada por atendimentos em saúde, sem uma efetividade organizacional almejada. Por consequência, tais fatores induzem o não seguimento do cuidado centrado na pessoa e em suas reais necessidades.^62 , 63^ Além das atividades administrativas e técnicas, torna-se essencial o investimento em ferramentas que otimizem o cuidado farmacêutico e promovam a inserção desse serviço de maneira equitativa em iniciativas emergentes, como o manejo de doenças crônicas não transmissíveis e a redução da mortalidade por essas condições.^62^

Devido à complexidade desse cenário, que envolve aspectos organizacionais dos serviços de saúde e a configuração profissional exigida para adequação nesse contexto, a priorização de disponibilidade inicial do cuidado farmacêutico para enfermos em estágio de alto risco pode melhorar o gerenciamento dos procedimentos de trabalho e beneficiar os pacientes com necessidades imediatas graves.^64^

A realidade dos farmacêuticos que trabalham com dispensação de medicamentos foi considerada para o desenvolvimento dessa ferramenta, uma vez que esses profissionais poderão utilizar o INSAF-HAS em sua rotina de trabalho. Diante da ampla gama de atividades realizadas (atendimento ao paciente, aquisição de medicamento, controle de estoque, entre outras incumbências administrativas e gerenciais), optou-se por desenvolver um formulário simples e prático, visto que o tempo disponível para o cuidado farmacêutico pode ser limitado. Assim, não seria viável a inclusão de um procedimento complexo e moroso nas atribuições profissionais. Além disso, o INSAF-HAS baseia-se em fatores determinantes na progressão clínica da HAS e que podem ser avaliados durante um contato rápido com o paciente, por exemplo, na dispensação do medicamento, tornando-se importante aliado na triagem de pacientes para o cuidado farmacêutico, podendo ser aplicado, inclusive, pelos auxiliares/técnicos de farmácia.

Dentre os fatores analisados para compor o instrumento, a PA elevada é um importante fator de risco no desenvolvimento e/ou agravamento das doenças cardiovasculares.^36 , 37 , 42 , 65 , 66^ Entretanto, aferi-la poderia limitar a utilização do instrumento por parte dos farmacêuticos e da equipe de farmácia, devido ao tempo necessário para realizar esse procedimento. Então, os especialistas discutiram a possibilidade de incluir uma questão sobre o controle pressórico em substituição da aferição da PA. A frequência com que o sujeito afere a PA pode ser inadequada, implicando no desconhecimento desses indivíduos sobre o controle da doença, e essa pergunta poderia conduzir a um viés de seleção dos pacientes. Por esse motivo, nenhuma questão foi incluída sobre o monitoramento dos níveis pressóricos, porém vale ressaltar que a ferramenta destina-se a pacientes com HAS, o que, por si só, já é um fator de risco para doenças cardiovasculares.^40 , 67^ O consenso final dos especialistas sobre isso, com o intuito de aprimorar o conteúdo e a aplicabilidade, foi acrescentar um item sobre o tratamento da PA.

Outro fator de risco para doenças cardiovasculares importante é o nível sérico elevado de colesterol (total e frações),^42 , 50^ cuja dosagem, por meio de exame laboratorial no serviço público brasileiro, é realizada aproximadamente a cada 6 meses. A avaliação ideal deste indicador seria por esses exames laboratoriais, mas as restrições referentes ao tempo e a recursos poderiam inviabilizá-lo, partindo da premissa de que seria necessária a busca de resultados (que poderiam estar desatualizados) em prontuários médicos ou a solicitação de novos exames. Assim, considerou-se, como melhor opção, questionar o paciente sobre o diagnóstico de dislipidemia e, em caso afirmativo, interrogá-lo sobre o tratamento conduzido, que também seria confirmado por meio da prescrição médica apresentada.

*Diabetes mellitus* também é um fator de risco para doenças cardiovasculares,^37 , 42 , 51 , 52^ e foi avaliado da mesma maneira que a dislipidemia. Ao invés de utilizar resultados de exames (glicemia ou hemoglobina glicada) ou teste de glicemia capilar, realizou-se questionamento sobre essa enfermidade e verificação na prescrição, referente à utilização de medicamentos antidiabéticos orais ou insulina. Com isso, teve-se a intenção de garantir a acessibilidade e a praticidade do INSAF-HAS.

O tabagismo configura-se como um fator de risco para doenças cardiovasculares,^36 , 37 , 40 , 42 , 43^ e foi acrescentada ao INSAF-HAS uma pergunta direta ao paciente: “Você foi ou é tabagista (fumante)?”. Nesta questão, não foi descrita apenas a palavra “fumante”, devido à experiência dos especialistas acerca da associação da expressão com o uso de drogas ilícitas (por exemplo, maconha e craque), pois essas também são “fumadas”. Como a palavra “tabagista” não é parte do vocabulário de muitas pessoas, especialmente de pessoas com menor escolaridade, optou-se por utilizar os dois termos.

Apesar de alguns estudos indicarem a ação benéfica de pequenas doses de bebida alcoólica, especialmente vinho tinto na aterosclerose,^68 , 69^ sabe-se que o consumo de álcool está relacionado com aumento de risco para HAS e deve ser evitado.^44 , 45^ De acordo com as Diretrizes Brasileiras de Hipertensão, não é recomendada a ingestão diária superior a 30 g de álcool para homens e 15 g para mulheres.^36^ Portanto, a averiguação desse consumo foi inserida no INSAF-HAS.

Outros fatores importantes, incluídos no instrumento, são a idade e o sexo do paciente.^36 - 38^ Indivíduos do sexo masculino apresentam mais chances de desenvolver doença cardiovascular e a ameaça torna-se maior a partir de 55 anos de idade. No caso das mulheres, o risco aumenta com o início da menopausa (aproximadamente 50 anos) e se eleva ainda mais após os 65 anos.

Enquanto a prática de exercício físico reduz o risco de ocorrência de doença cardiovascular, o sedentarismo é um fator de predisposição para a enfermidade.^36 , 40 , 41^ Dessa maneira, a ferramenta proposta por este estudo investiga a prática de exercício físico, que foi considerada como a realização de atividade física por pelo menos 30 minutos, três vezes na semana (90 minutos/semana).

O histórico familiar de HAS, dislipidemia, DM e agravamentos, como IAM e AVC em parentes de primeiro grau, também são fatores de risco para doenças cardiovasculares.^54 , 55^ Por isso, acrescentou-se uma questão sobre o histórico familiar no INSAF-HAS.

A manifestação prévia no paciente de agravamentos relacionados à HAS, também é um aspecto importante a ser considerado. Dentre os principais, pode-se citar: AVC, IAM, insuficiência renal e retinopatia hipertensiva.^37 , 53 , 70^ Dada a relevância desses fatores, mostrou-se importante acrescentá-los ao INSAF-HAS, entretanto, durante o pré-teste, percebeu-se que alguns sujeitos não sabiam informar com precisão a ocorrência desses agravamentos.

No caso da insuficiência renal, esse termo não foi compreendido por alguns pacientes, então realizou-se uma tentativa de utilizar a expressão “problema nos rins”, mas a incompreensão não pôde ser solucionada com essa mudança. Quando o indivíduo era questionado se “teve algum problema nos rins”, ele não foi capaz de diferenciar a insuficiência renal de outras doenças do sistema urinário, como cistite ou nefrolitíase. Devido à opção de não utilizar o prontuário médico, a confirmação do diagnóstico de insuficiência renal apenas pelo relato do sujeito revelou-se inefetiva. Por consequência, optou-se por não acrescentar ao instrumento final uma questão sobre esse fator de risco para doenças cardiovasculares.

Da mesma maneira, o termo “retinopatia hipertensiva” não foi compreensível para a maior parte dos participantes incluídos no pré-teste. Inicialmente substitui-se o termo por “problemas de visão”, mas distúrbios associados à miopia e à catarata levavam o paciente a responder “sim”, assim como ao ser interrogado sobre “qual o problema de visão” que possuía, o indivíduo também não era capaz de garantir uma resposta precisa. Devido à impossibilidade da confirmação do diagnóstico e para minimizar o risco de erro, esse item foi retirado da versão final do INSAF-HAS.

Ao contrário dos outros dois agravamentos citados anteriormente, o AVC e o IAM foram mais facilmente relatados pelos pacientes, que não apresentavam dúvidas quando questionados: “Você teve infarto?” e “Você teve acidente vascular cerebral (AVC) ou derrame”? Por isso, esses dois fatores permaneceram na composição do instrumento.

No que se refere à farmacoterapia, a adesão inapropriada ao tratamento anti-hipertensivo prescrito está relacionada ao controle inadequado da PA.^46^ Foram avaliados, então, diversos aspectos que podem afetar o seguimento do plano de cuidados, como escolaridade (quanto menor, maior a dificuldade do sujeito compreender a prescrição e, consequentemente, medicar-se corretamente), acesso ao medicamento, autonomia no momento de se medicar e polifarmácia.^39 , 47^ O indivíduo também foi questionado diretamente sobre a adesão, ou seja, se às vezes esquece ou deixa de utilizar os medicamentos prescritos.

Fatores cujo efeito associado à HAS ainda é controverso, como estresse,^67 , 71^ depressão^67 , 72^ e consumo de café,^36 , 73^ não foram incluídos. A obesidade e a ingestão de sal^36 , 40 , 74 - 76^ também foram consideradas, mas ponderou-se que essas avaliações debilitariam o uso da ferramenta. No caso da obesidade, a principal limitação é a do tempo, uma vez que seria necessário pesar e medir a altura do paciente e, com esses resultados, realizar o cálculo do índice de massa corporal. Por sua vez, é muito difícil de ser avaliada a quantidade de sal ingerido diariamente, pois a presença de sódio nos alimentos não se atém ao uso do sal de cozinha e, normalmente, as pessoas não sabem quanto dessa substância consomem por dia.

### Limitações e pontos fortes do estudo

Devido aos atributos almejados no desenvolvimento da ferramenta (acessibilidade e viabilidade de aplicação na rotina da farmácia), a maneira de avaliação de alguns fatores de risco para doenças cardiovasculares não apresentou esse perfil e, portanto, essas condições não foram inseridas na versão final. Contudo, enfatiza-se que o INSAF-HAS é um instrumento de triagem e, após a seleção dos pacientes, estes devem ser atendidos pelo serviço do cuidado farmacêutico. Desse modo, esses fatores que não foram incluídos na ferramenta podem ser avaliados durante esse processo.

Os especialistas que constituíram o painel de *experts* , para avaliação do INSAF-HAS pertenciam todos à Faculdade de Ciências Farmacêuticas de Ribeirão Preto da Universidade de São Paulo, sendo esta uma das limitações do presente trabalho.

Ademais, após apresentação detalhada da seleção dos itens, descrição da população-alvo e realização do piloto, observa-se que o INSAF-HAS possui validade de face e conteúdo. Entretanto, entende-se a necessidade de avaliação das propriedades psicométricas, que devem ser realizadas em estudos futuros.

Apesar dessas limitações, fica claro que esse estudo inicial pode colaborar na estruturação de serviços de cuidado farmacêutico e sugere-se que a utilização do INSAF-HAS identifique os pacientes mais necessitados e proporcione a otimização da assistência oferecida pelos profissionais. O estudo pode ainda contribuir para promover debates sobre o assunto, assim como fortalecer a prática do cuidado farmacêutico no Brasil.

## CONCLUSÃO

A ferramenta elaborada INSAF-HAS apresenta validade de face e conteúdo, tal como relaciona e pontua fatores de risco associados à hipertensão arterial sistêmica em indivíduos portadores dessa enfermidade. Sugere-se o auxílio da ferramenta na seleção de pacientes com maior necessidade de participação em um serviço de cuidado farmacêutico.
